# Pilomatricome: étude de 22 cas

**DOI:** 10.11604/pamj.2016.23.254.8674

**Published:** 2016-04-28

**Authors:** Fatima Zahra Nasreddine, Fouzia Hali, Soumiya Chiheb

**Affiliations:** 1 Service de Dermatologie CHU Ibn Rochd, Casablanca, Maroc

**Keywords:** Pilomatricome, épithélioma momifié de Malherbe, bras, Pilimatrixoma, calcified epithelioma of Malherbe, arm

## Abstract

Le pilomatricome est une tumeur cutanée fréquente et bénigne du follicule pileux chez l'enfant. C'est une tumeur annexielle souvent méconnue et confondue avec d'autres lésions cutanées. Les localisations habituelles sont la tête et le cou. Le but de ce travail est de rapporter une série de 22 cas comportant des formes inhabituelles colligées au service de dermatologie sur une période allant de Janvier 2006 jusqu'au Mai 2015. L’étude a concerné 16 femmes et 6 hommes. La moyenne d’âge était de 23,3 ans (4-80 ans). La localisation cervico faciale a été observée dans 12 cas, 2 patients avaient des localisations multiples, un garçon de 4ans avait une localisation au niveau fronto-temporal et une fillette de 14 ans avait une localisation au niveau du visage et de l'avant-bras, et un patient de 48 ans avait une localisation sous unguéale. L'aspect clinique était typique dans tous les cas avec des nodules sous cutanés de consistance pierreuse. Tous les patients ont bénéficié d'une exérèse des nodules sous anesthésie locale. L’étude histologique était en faveur d'un épithélioma momifié de Malherbe d'exérèse complète sans signes de malignité. Aucun patient n'a présenté de rechute. L'originalité de notre étude réside dans la présence de localisations exceptionnelles au niveau latéro-vertébral, des membres et sous-unguéale, l’âge de survenue inhabituel à 80 ans et la présence de localisations multiples signalées chez 2 enfants.

## Abstract (en)

Pilomatricoma is a common and benign skin tumor of childhood arising from the hair follicle matrix. It is an often misunderstood adnexal tumor, confused with other skin lesions. The most common sites of pilomatricome are the head and the neck. The aim of this study was to report a series of 22 cases with unusual forms collected in the dermatology department from January 2006 to May 2015. The study included 16 women and 6 men. The average age was 23.3 years (4- 80 years). Cervicofacial location was observed in 12 cases, 2 patients had multiple locations, a 4 year-old boy had frontotemporal location, a 14 year-old girl had face and forearm location and a 48 year-old patient had subungual location. Clinical features were typical in all cases, with subcutaneous nodules with a stony-hard consistency. All patients underwent excision of nodules under local anesthesia. Histological study supported a completely excised Malherbe's mummified tumor without malignancy. No patients relapsed. The originality of our study lies in the presence of exceptional locations in laterovertebral region, on limbs and in the subungual region and of unusual age of onset (80 year-old) as well as the presence of multiple tumor locations reported in 2 children.

## Introduction

Le pilomatricome ou épithélioma calcifié de Malherbe est une tumeur cutanée annexielle développée aux dépens de la matrice du poil. C'est la plus fréquente des tumeurs du follicule pileux rencontrée le plus souvent chez l'enfant. Les localisations les plus fréquentes sont la tête et le cou, l'atteinte des membres reste exceptionnelle. Le but de ce travail est de rapporter une série de 22 cas comportant des formes inhabituelles.

## Méthodes

Il s'agit d'une étude descriptive colligeant tous les cas de pilomatricomes suivis dans le service de dermatologie du CHU Ibn Rochd de Casablanca, entre Janvier 2006 et Mai 2015. Le diagnostic de pilomatricome a été confirmé par l'analyse histologique de la pièce opératoire. Pour chaque patient, nous avons précisé le sexe, l’âge le siège, les données épidémio-cliniques, le traitement et l’évolution.

## Résultats

**Figure 1:**
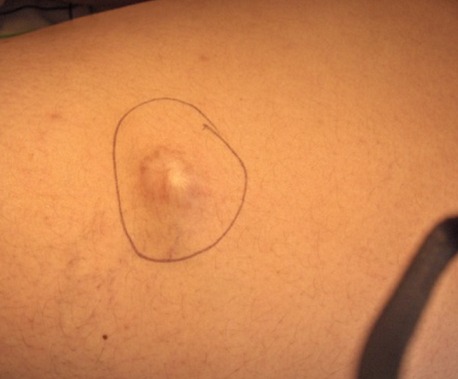
Localisation des members

**Figure 2:**
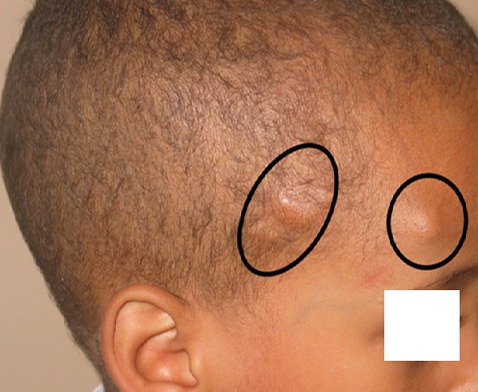
Localisation fronto-temporale

**Figure 3:**
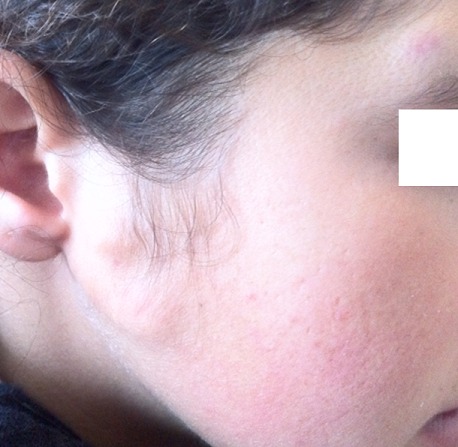
Localisation multiples au niveau du visage

**Figure 4:**
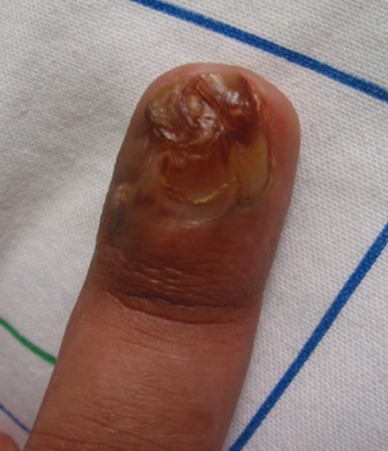
Pilomatricome sous unguéal

**Figure 5:**
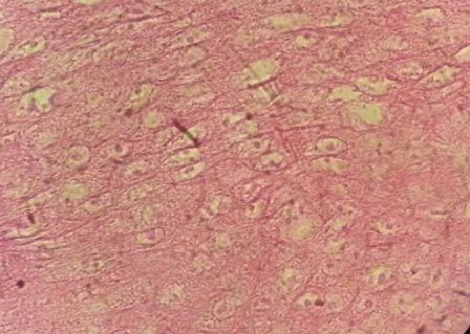
Aspect histologique

**Figure 6:**
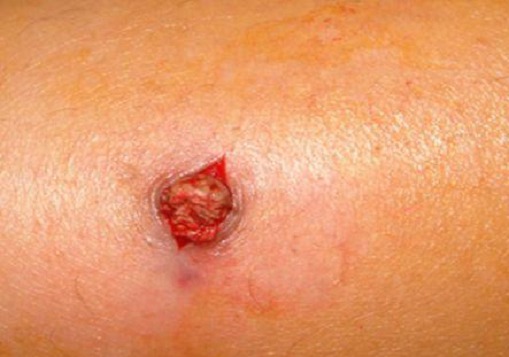
Excision chirurgicale

**Figure 7:**
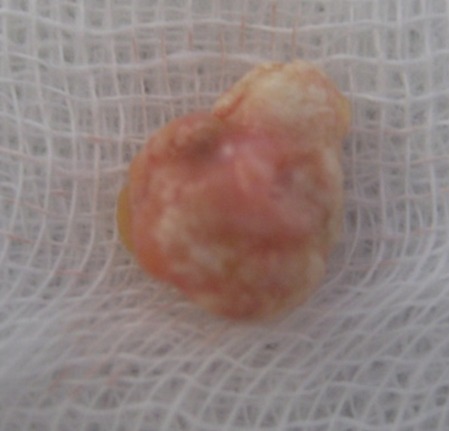
Aspect macroscopique après excision chirurgicale

Vingt deux cas ont été colligés avec un âge moyen de 23,3 ans (extrêmes de 4 à 80 ans) et une prédominance féminine (sexe ratio de 2,6 femmes pour un homme). L'atteinte de la région cervico-faciale prédominait dans 57% des cas. Les autres localisations étaient représentées par les membres dans 43% des cas (Figure 1), la région latéro vertébrale dans 1 cas. Deux patients avaient des localisations multiples, un garçon de 4 ans avait une localisation fronto- temporale (Figure 2), et une fillette de 14 ans avait une localisation au niveau du visage et l'avant bras (Figure 3) et un patient de 48 ans avait une localisation sous unguéale (Figure 4). L'aspect clinique typique était sous forme de nodules sous cutanés, de consistance dure indolore et fixe par rapport au plan profond avec une peau normale en regard. Le diagnostic de pilomatricome a toujours été suspecté cliniquement. Aucun examen d'imagerie complémentaire à visée diagnostique n'avait été réalisé en préopératoire. Mais l'analyse histologique avait confirmé dans tous les cas le diagnostic de pilomatricome devant des ilots de cellules momifiées entourées de travées osseuses avec foyers de calcifications (Figure 5). Aucune association clinique morbide n'avait été retrouvée chez ces patients porteurs de pilomatricome. Une excision chirurgicale emportant un fuseau cutané en regard de la lésion (Figure 6, Figure 7) a été réalisée chez tous les patients. Aucune récidive n'a été rapportée avec un recul moyen de 4 ans.

## Discussion

Le pilomatricome a été décrit par Malherbe Chenantais en 1880 comme une tumeur bénigne, calcifiée des glandes sébacées, et son origine été confirmée plus tard par Forbis et Helwing [1, 2]. En effet ces deux auteurs ont démontré par le biais d'une étude immunohistochimique que le point de départ de la tumeur était les cellules de la matrice pilaire. Le pilomatricome se présente typiquement sous la forme d'un nodule sous-cutané asymptomatique rond ou ovalaire irrégulier, de consistance dure ou ferme. La peau en regard de la lésion est souvent bleutée. La tumeur adhère au plan superficiel, alors qu'elle est mobile par rapport aux plans profonds. Le signe de la tente décrit par Graham et Meruim sans être pathognomique est très évocateur du diagnostic [2]. Le pilomatricome comporte à l'inspection une ou plusieurs faces planes séparées entre elles, par des lignes angulaires donnant l'impression d'une tente. Ce signe est souvent mis en évidence en étirant la peau et en recherchant l'existence d'angles ou de facettes. Il pourrait être en rapport avec la quantité de calcium déposé dans la tumeur. Le pilomatricome peut revêtir différentes formes cliniques et être perforant, ulcéré, anétodermique avec une peau érythémateuse en regard de la lésion ou pigmentée [1, 2]. Ce qui explique les erreurs diagnostiques retrouvées dans la littérature. Chez nos patients, la peau en regard était normale. Dans la plupart des cas publiés, le diagnostic préopératoire est évoqué dans seulement un tiers des cas [3]. La difficulté du diagnostic clinique repose sur l'aspect clinique variable du pilomatricome et sur la méconnaissance de cette tumeur par certains cliniciens. Certains auteurs ont essayé d'améliorer les moyens du diagnostic clinique du pilomatricome par dermoscopie cependant ceci n'est pas suffisant pour le diagnostic de certitude [4]. Le diagnostic de pilomatricome doit rester clinique, confirmé par l'histologie qui permet d’éliminer certains diagnostics différentiels principalement les kystes épidermoides et pilaires mais surtout le pilomatricome malin. L’étude immunohistochimique facilite la distinction [3]. La dégénérescence carcinomateuse du pilomatricome reste controversée [5, 6]. Certains auteurs ont toutefois proposé des examens d'imagerie complémentaires comme l’échographie pour aider au diagnostic. L'IRM a peu d'intérêt en pédiatrie [7, 8]. La radiographie standard est utile uniquement devant la suspicion d'un pilomatricome significativement calcifié. Cependant dans la plupart des cas le pilomatricome était diagnostiqué en post opératoire. La plupart des auteurs rapportent la survenue du pilomatricome chez l'enfant particulièrement avant dix ans [2, 9]. Cependant il peut apparaitre à tout âge, des formes congénitales ont été également rapportées [10]. Dans notre série un patient avait 80 ans ce qui est inhabituel. Le pilomatricome est plus fréquent chez la femme dans la majorité des séries publiées avec un sex-ratio de 1,5 femme atteinte pour un homme [11]. Dans notre série le sexe-ratio était de 2,6 femmes pour un homme. En ce qui concerne les différentes localisations de pilomatricome, nos résultats sont en accord avec les données de la littérature avec une atteinte fréquente de la région cervico-faciale [12]. Seulement quelques exceptionnelles localisations au niveau des membres, ont été observées [7, 13]. Le pilomatricome est une tumeur généralement unique. Cependant certains patients développent de façon simultanée ou successive plusieurs pilomatricomes [3, 8]. Dans notre série deux patients présentaient deux localisations simultanées de pilomatricome. Le siège au niveau sous unguéale n'a pas été rapporté à notre connaissance dans la littérature. Nous rapportons le premier cas dans notre série. Aucune association morbide n'est rapportée dans notre série. Une association du pilomatricome avec la dystrophie myotonique de steinert a été retrouvée avec une prédominance de localisations multiple et de formes familiales [3]. Le traitement du pilomatricome est l'excision chirurgicale complète emportant un fuseau cutané, surtout si la lésion est adhérente au derme. Il s'agit du traitement de référence permettant d’éviter la majorité des récidives [11]. Le pronostic du pilomatricome est bon. La guérison sans récidive est la règle après exérèse chirurgicale totale.

## Conclusion

Le pilomatricome est une tumeur cutanée bénigne généralement unique. Le siège cervico-facial et le sexe féminin sont les caractéristiques habituelles. Les localisations multiples et l'atteinte des membres sont exceptionnelles.

### Etat des connaissance sur le sujet

- Le pilomatricome est une tumeur cutanée bénigne du follicule pileux, rare.
- Il survient principalement chez l'enfant avec prédominance féminine (sex-ratio de 3/2).
- Les localisations habituelles sont le cou et la tête et le caractère généralement unique de cette tumeur.

### Contribution de notre étude à la connaissance

- L'originalité de notre série réside dans la localisation exceptionnelle au niveau sous unguéal, Nous rapportons à notre connaissance le premier cas dans la littérature.
- L’âge de survenue inhabituel à 80ans.
- Le caractère multiple de cette tumeur chez 2 de nos patients ce qui est exceptionnelle.
